# Genetic Stability of Bacterial Artificial Chromosome-Derived Human Cytomegalovirus during Culture *In Vitro*

**DOI:** 10.1128/JVI.02858-15

**Published:** 2016-03-28

**Authors:** Isa Murrell, Gavin S. Wilkie, Andrew J. Davison, Evelina Statkute, Ceri A. Fielding, Peter Tomasec, Gavin W. G. Wilkinson, Richard J. Stanton

**Affiliations:** a Division of Infection and Immunity, School of Medicine, Cardiff University, Cardiff, United Kingdom; b MRC-University of Glasgow Centre for Virus Research, Glasgow, United Kingdom

## Abstract

Clinical human cytomegalovirus (HCMV) strains invariably mutate when propagated *in vitro*. Mutations in gene RL13 are selected in all cell types, whereas in fibroblasts mutants in the UL128 locus (UL128L; genes UL128, UL130, and UL131A) are also selected. In addition, sporadic mutations are selected elsewhere in the genome in all cell types. We sought to investigate conditions under which HCMV can be propagated without incurring genetic defects. Bacterial artificial chromosomes (BACs) provide a stable, genetically defined source of viral genome. Viruses were generated from BACs containing the genomes of strains TR, TB40, FIX, and Merlin, as well as from Merlin-BAC recombinants containing variant nucleotides in UL128L from TB40-BAC4 or FIX-BAC. Propagation of viruses derived from TR-BAC, TB40-BAC4, and FIX-BAC in either fibroblast or epithelial cells was associated with the generation of defects around the prokaryotic vector, which is retained in the unique short (U_S_) region of viruses. This was not observed for Merlin-BAC, from which the vector is excised in derived viruses; however, propagation in epithelial cells was consistently associated with mutations in the unique long *b*′ (U_L_/*b*′) region, all impacting on gene UL141. Viruses derived from Merlin-BAC in fibroblasts had mutations in UL128L, but mutations occurred less frequently with recombinants containing UL128L nucleotides from TB40-BAC4 or FIX-BAC. Viruses derived from a Merlin-BAC derivative in which RL13 and UL128L were either mutated or repressed were remarkably stable in fibroblasts. Thus, HCMV containing a wild-type gene complement can be generated *in vitro* by deriving virus from a self-excising BAC in fibroblasts and repressing RL13 and UL128L.

**IMPORTANCE** Researchers should aim to study viruses that accurately represent the causative agents of disease. This is problematic for HCMV because clinical strains mutate rapidly when propagated *in vitro*, becoming less cell associated, altered in tropism, more susceptible to natural killer cells, and less pathogenic. Following isolation from clinical material, HCMV genomes can be stabilized by cloning into bacterial artificial chromosomes (BACs), and then virus is regenerated by DNA transfection. However, mutations can occur not only during isolation prior to BAC cloning but also when virus is regenerated. We have identified conditions under which BAC-derived viruses containing an intact, wild-type genome can be propagated *in vitro* with minimal risk of mutants being selected, enabling studies of viruses expressing the gene complement of a clinical strain. However, even under these optimized conditions, sporadic mutations can occur, highlighting the advisability of sequencing the HCMV stocks used in experiments.

## INTRODUCTION

Human cytomegalovirus (HCMV) is associated with morbidity and mortality in immunocompromised individuals and is the leading infectious cause of birth defects worldwide (1). As a result, the development of a vaccine capable of eliciting protection against congenital infection is recognized as a high priority (2). Antiviral therapy can be highly effective in controlling HCMV disease but is often confounded by toxicity and the rapid selection of resistance. Therefore, more effective therapeutic options to combat HCMV are needed urgently.

In developing novel therapies against a pathogen, researchers should ideally study strains that accurately represent the causative agent of disease. Unfortunately, HCMV strains propagated in cell culture reproducibly lose many of the properties characteristic of clinical virus. Serial passage of HCMV has been correlated with reduced virulence (3–6), altered tropism (7–10), decreased natural killer (NK) cell resistance (11), and loss of cell-associated growth (7, 10). These alterations are associated with genetic changes in the virus (7, 12–14). Indeed, a systematic study of HCMV clinical strains passaged *in vitro* showed that mutations were selected reproducibly in a subset of HCMV genes, most commonly causing protein truncation by nucleotide substitutions (resulting in in-frame stop codons) or small insertions or deletions (resulting in frameshifts) (12). Thus, regardless of whether HCMV clinical strains are passaged on fibroblast, epithelial, or endothelial cells, mutations tend to occur in gene RL13, which encodes a virion envelope glycoprotein affecting efficient cell-to-cell spread and the release of virus from cells (12, 15). When HCMV is propagated in fibroblasts, mutations are also selected in the UL128 locus (UL128L) (16), which encodes three proteins (pUL128, pUL130, and pUL131A). These proteins associate with glycoproteins H and L to form a pentameric complex that is required for efficient infection of epithelial, endothelial, and myeloid cells (17–22), but that selectively impedes infection in fibroblasts (12, 15, 20, 23, 24). Irrespective of cell type, additional mutations are often observed in the unique long *b*′ (U_L_/*b*′) region of the genome, which contains 20 genes, including the CXCL chemokine homologues UL146 and UL147 (25, 26), the NK cell evasion functions UL135, UL141, and UL142 (27–32), a tumor necrosis factor receptor homologue (UL144) (33, 34), and a cluster of genes implicated in controlling viral reactivation from latency as well as efficient infection of endothelial cells (UL138, UL136, UL135, and UL133) (35–40). Sporadic mutations occur elsewhere and gradually accumulate in strains subjected to prolonged passage (14, 41).

To preserve HCMV genomes they can be cloned into a bacterial artificial chromosome (BAC) (42, 43), and infectious virus can then be recovered by DNA transfection of permissive cells. In the strain Merlin-BAC, which was generated from a virus that had undergone only six passages in fibroblasts since isolation, the prokaryotic vector was inserted into the noncoding region between genes US28 and US29 (15). By virtue of the presence of flanking *loxP* sites and an internal *cre* gene, the vector is self-excising so that virus generated from the BAC differs from the original virus in this region only by a residual insertion of 40 bp. Mutations in RL13 and UL128 that were acquired *in vitro* prior to BAC cloning were repaired so that the final clone (Merlin-BAC) contains the wild-type (wt) HCMV gene complement. Comparison of the sequences of Merlin-BAC and Merlin in the original clinical sample showed that their HCMV components are virtually identical (44). However, when virus derived from the Merlin-BAC was passaged in fibroblasts, novel mutations in RL13 and UL128L were selected, in a similar manner to selection in clinical HCMV strains (15).

Other frequently used BAC-cloned genomes include those captured from HCMV strains TR (TR-BAC) (45, 46), TB40/E (TB40-BAC4) (7, 47), and VR1814 (FIX-BAC) (9, 48). However, the most commonly used versions of these BAC-cloned strains are incapable of generating a virus containing the wild-type HCMV gene complement because the region containing genes US2, US3, and US6 has been replaced in each by a nonexcisable vector, and each has also suffered mutations during *in vitro* passage of the parental virus (23, 44, 49, 50). However, unlike clinical strains that have been passaged on fibroblasts, the protein-coding regions of UL128L in these clones appear to be intact. Furthermore, there have been no reports of mutations in UL128L being selected when viruses from these BAC-cloned strains are generated in fibroblasts. Previously, we investigated the properties of TR-BAC, TB40-BAC4, and FIX-BAC UL128L by transferring sequences from each into Merlin-BAC (23). We concluded that the UL128L genome region of TR-BAC carried no *in vitro*-acquired mutations; however, single nucleotides were identified within TB40-BAC4 UL128 (a G to T substitution [G>T] in an intron, affecting splicing) and FIX-BAC UL130 (A>G, causing an amino acid substitution) that were responsible for (i) reduced amounts of pentameric complex being present in the virion, (ii) increased cell-free titers in epithelial and fibroblast cells, and (iii) increased rates of cell-to-cell spread in fibroblasts but reduced rates in epithelial cells.

In the present study, we sought to investigate parameters that would support the propagation of clonal HCMV stocks that retained genomic integrity. HCMV BAC constructs containing strains Merlin, TR, FIX, and TB40/E provided a defined clonal source of each virus. We also characterized viruses in which Merlin-BAC UL128L had been replaced in its entirety by the corresponding region of TB40-BAC4 or FIX-BAC and those in which it had been modified by adding only the single nucleotide differences mentioned above (23). To identify conditions under which HCMV might be propagated without mutations being selected, viruses were derived from BACs and then passaged in epithelial or fibroblast cells, and the genome populations were deep sequenced. The frequency with which mutations (including sizeable deletions) were found across all strains demonstrates the merits of performing HCMV genome sequencing as a routine quality control step during the production of virus stocks. Crucially, we showed that it is possible to produce HCMV stocks containing a stable, complete complement of wild-type genes by recovering virus in fibroblasts from a self-excising BAC in which expression of RL13 and UL128L is reversibly repressed.

## MATERIALS AND METHODS

### Cells and viruses.

The cell lines used included primary human fetal foreskin fibroblast (HFFF) cells, which were kindly supplied by Graham Farrar (CAMR, Salisbury, United Kingdom), and human telomerase reverse transcriptase (hTERT)-immortalized retinal pigmented epithelial cells (RPE-1) (ATCC CRL-4000). A cell line (HFFF-Tet) constitutively expressing the tetracycline (Tet) repressor was derived from hTERT-immortalized HFFFs (HFFF-hTERT) (51) transduced with the Tet repressor gene (*tetR*) (15). Cell lines were propagated at 37°C in 5% (vol/vol) CO_2_ in Dulbecco's modified Eagle's medium (DMEM) supplemented with 10% (vol/vol) fetal bovine serum, 100 U ml^−1^ penicillin, and 100 μg ml^−1^ streptomycin.

Several of the BAC-cloned HCMV strains and derived variants used in this study have been described previously (Table 1). Merlin-BAC contains the full complement of wild-type genes, and the variant BACs used were identical in sequence to it except in RL13 and UL128L. Thus, each variant contained the same frameshift mutation in RL13 that had been selected during propagation of the parental virus *in vitro*, and each also differed in UL128L. In addition, each variant was engineered to express enhanced green fluorescent protein (eGFP) from an internal ribosome entry site (IRES) inserted immediately downstream of gene UL122 (15). TR-BAC, TB40-BAC4, and FIX-BAC were kindly provided Jay Nelson (Oregon Health and Science University, Portland, OR, USA), Christian Sinzger (University of Tubingen, Germany), and Gabrielle Hahn (Universitätklinikum Carl Gustav Carus, Dresden, Germany), respectively. An IRES:eGFP cassette was also inserted after UL122 in these clones.

**TABLE 1:** BAC-cloned HCMV strains and derivatives used

HCMV strain	Name	GenBank accession no.	Reference	UL128L origin and nucleotide features^*b*^	Designation of derived virus
BAC-cloned strains^*a*^					
Merlin	Merlin-BAC	GU179001.1	15	Wild-type	Merlin-UL128L^wt^
				G>A in UL128 at nt 176260 (R>stop)	Merlin-UL128L^mut^
TR	TR-BAC	AC146906.1	45	Intact	TR
TB40/E	TB40-BAC4	EF999921.1	47	Intact	TB40
VR1814	FIX-BAC	AC146907.1	48	Intact	FIX
Clinical strain (nonpassaged)					
3301		GQ466044.1	41	Wild type	3301
Merlin derivatives					
Containing UL128L of other strains			23	3301	Merlin-UL128L^3301^
				TR	Merlin-UL128L^TR^
				TB40-BAC4	Merlin-UL128L^TB40^
				FIX	Merlin-UL128L^FIX^
Containing single nucleotides from UL128L of other strains			23	TB40-BAC4 G>T in UL128 at nt 176612 (intron)	Merlin-UL128^G>T^
				FIX-BAC A>G in UL130 at nt 177364 (S>P)	Merlin-UL130^A>G^

a GenBank accession numbers for the BAC-cloned parental strains are as follows: Merlin, NC_006273.2 (74); TR, KF021605.1 (46); VR1814, GU179289.1 (9). An accession number for strain TB40/E (7) is not available.

b Nucleotide positions are relative to the sequence of BAC-cloned HCMV strain Merlin (GenBank accession number GU179001). nt, nucleotide.

### *In silico* analysis of HCMV DNA sequences.

Sequence comparisons, the design of recombineering experiments, primer and oligonucleotide design, and sequence data analysis were performed by using CLC Main Workbench, version 6, software (CLC Bio). The attributes of primers and oligonucleotides were verified by using Oligo Explorer, version 1.4 Beta (GeneLink), and Oligo Analyzer (GeneLink) software. Primers and oligonucleotides were purchased from Sigma-Aldrich as reverse-phase, cartridge-purified, lyophilized DNA.

### Recombineering of HCMV genomes.

All recombineering was performed as described previously (15, 23, 52, 53). Briefly, recombineering was achieved by successive transformations of Escherichia coli SW102 cells containing the BACs. A selectable Amp^r^ *sacB lacZ* cassette was inserted into the region to be modified, followed by screening by positive selection on medium supplemented with ampicillin (50 μg ml^−1^). The selectable cassette was then replaced by the required DNA sequence, followed by negative selection on medium supplemented with sucrose (5%, wt/vol) to select against *sacB* expression and with 5-bromo-4-chloro-3-indolyl-β-d-galactopyranoside and isopropyl β-D-1-thiogalactopyranoside to identify colonies lacking *lacZ* expression.

Primers used to insert Tet operators upstream of RL13 and UL131A have been described previously (15). Primers to insert nucleotides from TB40-BAC4 and FIX-BAC into Merlin-BAC have also been described previously (23). Using primers as previously described (28), we also constructed a virus lacking UL141 (Merlin ΔUL141) in the background of Merlin-tetUL128^wt^, which contains binding sequences for the Tet operator inserted upstream of UL131A in the Merlin-BAC expressing wild-type UL128.

### Preparation of BAC DNA stocks.

BAC stocks were prepared by using a Nucleobond BAC 100 plasmid purification kit (Macherey-Nagel), according to the manufacturer's instructions, for the purification of low-copy-number plasmids. The concentration of purified plasmid DNA preparations was determined by using an ND1000 spectrophotometer (NanoDrop).

### Reconstitution of virus from BACs by transfection.

Viruses were reconstituted from BACs by transfection of 0.5 × 10^6^ epithelial cells, 2 × 10^6^ HFFF cells, or 2 × 10^6^ HFFF-Tet cells. HFFF and HFFF-Tet cells were transfected by electroporation, using program T16 of the Nucleofector II (Amaxa) and a basic Nucleofector kit (Lonza), according the manufacturer's instructions. On occasions when transfected HFFF or HFFF-Tet cultures were <70% confluent after overnight recovery, additional nontransfected cells were added. RPE-1 cells were transfected by chemical transfection using an Effectene kit (Qiagen), according to the manufacturer's instructions for transfection of adherent cells. The efficiencies of transfection of fibroblast and epithelial cells differed only marginally, with 20 to 35 plaques formed per electroporation in HFFF or HFFF-Tet cells and 50 to 60 plaques formed in RPE-1 cells.

### Preparation of viral stocks for sequencing from RPE-1 cells.

Following transfection, infected cultures were maintained until plaques were visible. In RPE-1 cell cultures, infected cells were then trypsinized weekly and reseeded into fresh flasks. As well as modeling the way in which viruses are commonly grown for experimental use, this also reduced the time until 100% cytopathic effect (CPE) was observed (minimizing the chance of mutants being selected) and reduced the selective pressure for selection of mutants that favor cell-free spread. This procedure was continued until 100% CPE was observed, at which point the cells were trypsinized and cocultured with 6 × 10^6^ uninfected RPE-1 cells. Cultures were again trypsinized weekly until 100% CPE was observed, and then the infected cells were cocultured with 3 × 10^7^ uninfected RPE-1 cells. When 100% CPE was observed, virus was harvested from the supernatant every 2 days and stored at −80°C. When complete lysis had occurred, the supernatants were thawed, pooled, and cleared by centrifugation at 1,200 × *g* for 3 min, and then virus was pelleted at 30,000 × *g* for 2 h before being resuspended in DMEM–10% fetal calf serum (FCS).

### Preparation of viral stocks for sequencing from HFFF cells.

In HFFF cells, a protocol of weekly trypsinization, as used in RPE-1 cells, was employed for viruses that spread predominantly by the cell-to-cell route. As indicated above, this was done in order to model the way in which viruses are commonly grown for experimental use and to minimize the chance of mutants being selected by reducing both the time until 100% CPE was observed and the selective pressure for mutants that favored cell-free spread. For viruses that already spread efficiently by the cell-free route, cultures were maintained without splitting. When 100% CPE was observed, virus was harvested from the supernatant, concentrated as described above, and titrated by immunostaining the HCMV IE1 protein (see below). Uninfected HFFF cells were then infected at a multiplicity of infection (MOI) of 0.05, and virus was again allowed to spread through the monolayer until 100% CPE was observed, at which point virus was harvested and titrated. This procedure was repeated five times.

### Preparation of viral stocks from HFFF-Tet cells for sequencing.

In HFFF-Tet cells, all viruses spread by the cell-free route. Cultures were maintained until 100% CPE was observed, and then cell-free virus was used to infect 3 × 10^7^ uninfected cells. When 100% CPE was observed, virus was harvested every 2 days before being pooled and concentrated as described above.

### Quantification of infectivity.

Infectivity was quantified by titration in HFFF cells, which are the only cells that supported productive infection by all the HCMV strains and variants used. Viruses were quantified by plaque titration assay in which cells were inoculated with a range of 10-fold dilutions prepared from each viral stock and then incubated under an overlay composed of a mixture (1:1) of 2× DMEM and Avicel, which limited viral dissemination to the cell-to-cell route (54). Two weeks later, the overlay was removed, the plate was washed with phosphate-buffered saline (PBS), and plaques were counted. Alternatively, titration was performed by immunostaining the HCMV IE1 protein.

For immunostaining, cells grown in clear-bottomed, 96-well plates were infected, and, after 12 to 24 h of incubation, they were fixed in 4% (wt/vol) paraformaldehyde and then permeabilized with PBS containing 0.1% (vol/vol) Triton X-100. All treatments of cells were performed for 10 min at room temperature and followed by three washes in excess PBS. The primary antibody was a monoclonal mouse anti-IE1 antibody (MA1-7596; ThermoScientific) diluted (1:1,000) in PBS and incubated for 1 h at 37°C. Samples were washed in excess PBS and then incubated with Alexa Fluor 594-conjugated goat anti-mouse antibody F(ab′)2 fragment (A11020; Invitrogen) diluted (1:500) in PBS for 30 min at 37°C. Finally, the cells were washed in PBS, and fluorescently stained nuclei were counted.

### Viral genomic DNA extraction.

In initial experiments with virus derived from RPE-1 cells, there was a concern that the levels of viral DNA in virions would not be sufficient for sequencing. Therefore, viral genomic DNA was amplified by infecting HFFF cells at a high MOI and harvesting DNA at 3 days postinfection, conditions under which there was little chance of mutations occurring, and only small differences in the relative ratios of any mutant genomes were anticipated. Viral DNA was harvested from infected cells by using a DNeasy blood and tissue kit (Qiagen).

For the two viruses (Merlin-UL128L^wt^ and Merlin expressing UL128L from unpassaged strain 3301 [Merlin-UL128L^3301^]) (Table 1) for which titers were insufficient to perform infections at high MOIs, viral genomic DNA was extracted directly from 100 μl of cell-free virus stock by using a MinElute virus spin kit (Qiagen) according to the manufacturer's instructions. This method proved successful and was thereafter used for sequencing of all viruses, including all those from HFFF and HFFF-Tet cells.

### High-throughput sequencing of HCMV genomes.

Sequence read data sets were generated from viral genomic DNA by using a MiSeq system (Illumina, San Diego, CA, USA). DNA (1,000 ng) from each sample was sheared by sonication, and DNA fragments were purified and size selected by using AMPure XP beads (Beckman-Coulter, High Wycombe, United Kingdom). The DNA fragments were end repaired, 3′ adenylated, ligated to paired-end multiplexing adapters, and amplified by PCR using standard methods (Illumina). The data sets were filtered to remove nucleotides with a phred quality of <30 and processed for adapter removal by using Trim Galore, version 0.2.2 (http://www.bioinformatics.babraham.ac.uk/projects/trim_galore). The filtered reads were aligned against reference sequences by using the Burrows-Wheeler Aligner (BWA), version 0.6.2-r126 (55). Indexed bam files were generated from the alignments by using Samtools, version 0.1.18 (55), and viewed manually throughout using Tablet, version 1.12.12.05, in order to highlight any differences (56). Genome coverage ranged from 99.855 to 100%, average depth ranged from 117.841 to 769.865 reads/nucleotide.

### Sanger DNA sequencing.

When high-throughput sequencing identified mutations in viruses passaged in HFFF cells, Sanger sequencing was used to determine at which passage the mutations became detectable. The appropriate region of the genome was PCR amplified from viral genomic DNA isolated from each passage. PCR products were then sequenced by Sanger sequencing (Eurofins MWG). Primers used for PCR and sequencing reactions are listed in Table S1 in the supplemental material. Sequence data were analyzed using the CLC Main Workbench and compared with those derived by high-throughput sequencing.

## RESULTS

### Stability of BAC-derived HCMV strains in epithelial cells.

A major aim of the present study was to identify conditions that would permit the genetic integrity of low-passage-number HCMV strains to be retained during the preparation of viral stocks. UL128L is problematic because it impedes growth in fibroblasts, whereas it is required for the efficient infection of epithelial, endothelial, and myeloid cells (12, 15, 16). Several different HCMV BAC constructs (Table 1) were therefore transfected into epithelial (RPE-1) cells, with the aim of keeping UL128L functionally intact. Viruses derived from TR-BAC, TB40-BAC4, FIX-BAC, and Merlin-BAC were analyzed, as well as several from variant Merlin BACs (23). These variants included the following: two with wild-type UL128L, from either Merlin (Merlin-UL128L^wt^) or the unpassaged strain 3301 (Merlin-UL128L^3301^), which incorporate high levels of the pentameric complex into virions; one unable to synthesize the pentameric complex (Merlin-UL128L^mut^); two containing sequences from TB40-BAC4 (either the entire UL128L [Merlin-UL128L^TB40^] or a previously identified single nucleotide variation in UL128 [Merlin-UL128^G>T^]); and two containing sequences from FIX-BAC (either the entire UL128L [Merlin-UL128L^FIX^] or a single nucleotide variation in UL130 [Merlin-UL130^A>G^]). For the last four constructs, we have shown that the levels of the pentameric complex incorporated into virions are reduced relative to levels in wild-type Merlin (23). Merlin-UL128L^mut^ and FIX do not grow in RPE-1 cells (23), but viruses were generated from all other BAC constructs, expanded until 3 × 10^7^ cells were infected, harvested, and then sequenced.

**FIG 1:**
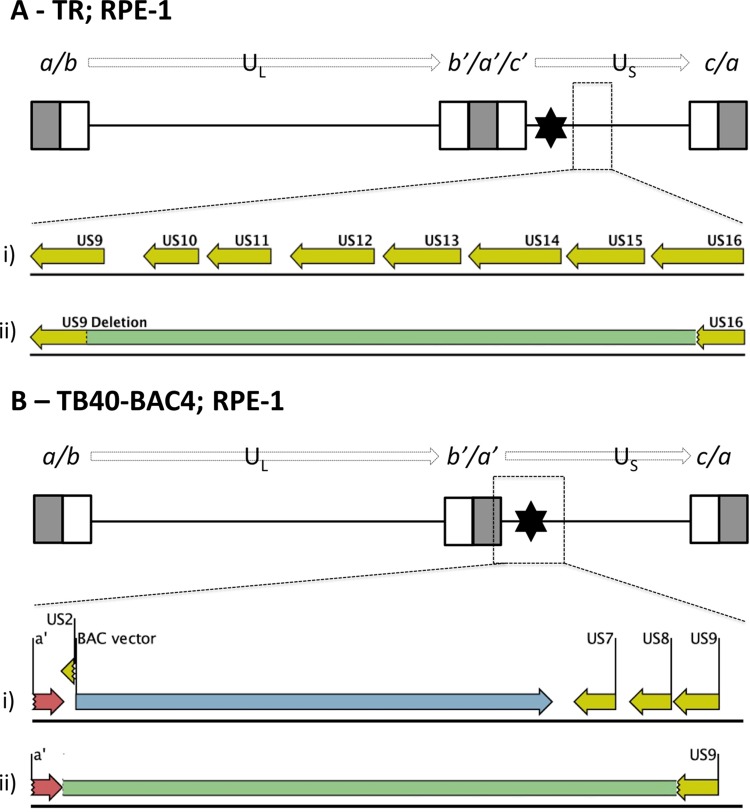
Mutant populations detected in stocks of virus derived from TR-BAC and TB40-BAC4 in RPE-1 cells. (A) TR. The top of the panel shows a schematic of TR-BAC. Enlarged boxes represent sequences repeated at each genome segment terminus (*a* sequences in gray; *b* and *c* sequences in white); the connecting black line represents regions of unique sequence, U_L_ and U_S_; the black star indicates the site of the stably incorporated BAC vector insertion. ORFs US9 to US16 in the parental TR BAC are shown (i), and sequence deleted (green) in the mutated TR genomes, originating from within US9 and spanning ORFs up to and including part of US16, is indicated (ii). (B) TB40-BAC4. The top of the panel shows a schematic of TB40-BAC4. The *a*′-US9 region in the parental TB40-BAC4, including the *a*′ sequence (red) fused to the remainder of ORF US2, the BAC vector (blue), US7, US8, and US9, is shown (i). Sequence deleted (green) in the mutated TB40-BAC4 genome, originating from the *a*′ terminus up to and including part of US9, is indicated (ii).

Although UL128L remained intact in all but one virus propagated in RPE-1 cells, mutations elsewhere were evident in all stocks (Table 2). By comparing all viruses, two consistent patterns of mutation were noted. First, TR-BAC and TB40-BAC4 are designed to retain the vector in progeny virus, and this feature appeared to have an impact on the stability of the viral genome in that HCMV sequences either within or adjacent to the vector were deleted in viruses derived from both BACs. The major TR population had a deletion from US9 to US16, supplemented by a frameshift mutation in UL84 (Fig. 1A and Table 2). The major TB40-BAC4 population had lost the entire vector sequence together with flanking HCMV sequences encoding residual portions of US2 and US7-US9 (Fig. 1B) and also had a premature stop codon in UL84.

**TABLE 2:** Mutant populations in viruses generated in epithelial (RPE-1) cells

HCMV strain or derivative	Location of mutation (nt)^*a*^	ORF(s) affected	Description of mutation and effect on coding	Mutant population (% of sequenced population)
TR	3687–9738	US9–US16	6,052-bp deletion	81
	159574–159581	UL84	TTTTTTTT>TTTTTTTTT (frameshift)	77
TB40	228878–1973	IRS1–US9	Deletion of 173 bp (end of *a*′ to US2), entire BAC vector, and 1,973 bp (US7, US8, and part of US9)	87
	155175	UL84	G>A (Q>stop)	46
Merlin-UL128L^wt^	176129–188023	UL128–UL136	11,895-bp deletion with 1,21- bp insertion (part of UL20 flanked by E. coli sequences)	3
Merlin-UL128L^3301^	180563–194029	UL147–UL150	∼13,750-bp deletion with 10,300-bp insertion (inversion of left genome end to within RL12)	44
Merlin-UL128L^TB40^	181910–191511	UL145–UL148C	9,602-bp deletion with 3,733-bp insertion (E. coli)	93
	183591–186632	UL142–UL139	3,042-bp deletion	6
Merlin-UL128^G>T^	32652	UL26	G>T (R>S)	48
	110792	UL75	G>C (L>M)	40
	185141	UL141	C>T (W>stop)	64
	204004	US8	A>C (V>G)	19
Merlin-UL128L^FIX^	184459–184914	UL141	456-bp in-frame deletion	100

a Locations are based on the sequences of TR-BAC (GenBank accession number AC146906.1), TB40-BAC4 (GenBank EF999921.1), and Merlin-BAC (GenBank GU179001.1). The TR-BAC, TB40-BAC4, and FIX-BAC sequences deposited in GenBank commence immediately following the site of vector insertion, and coordinates of mutations in viruses derived from these clones describe sequences either side of the BAC vector. nt, nucleotide.

**FIG 2:**
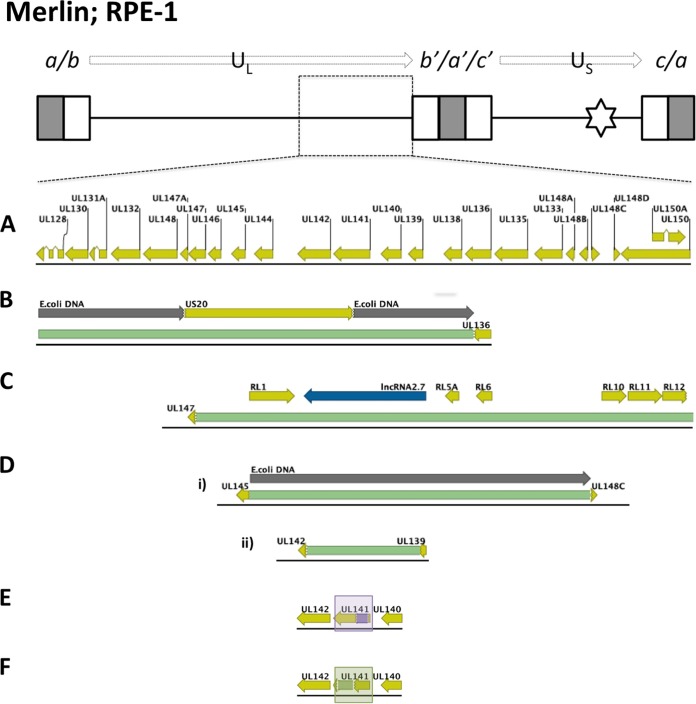
Mutant populations detected in stocks of Merlin viruses produced in RPE-1 epithelial cells. At the very top of the figure a schematic of the Merlin-BAC genome is shown. Enlarged boxes represent sequences repeated at each genome segment terminus (*a* sequences in gray; *b* and *c* sequences in white); connecting black lines represent unique sequence U_L_ and U_S_ genome regions; the star indicates the site of self-excising BAC vector insertion in the US28-US29 intergenic region. (A) Organization of ORFs UL128 to UL150 in the parental Merlin-BAC. (B) In Merlin-UL128L^wt^, mutant genomes underwent deletion (green) of sequence spanning UL128 to UL138 and part of UL136, with concomitant insertion of E. coli DNA (dark gray) and duplication of part of US20. (C) In Merlin-UL128L^3301^, mutant genomes underwent deletion of sequence from within UL147, up to and including UL150, with concomitant insertion of duplicated sequence (U_L_-start to RL12). (D) In Merlin-UL128L^TB40^, two mutant genomes were detected: (i) a major population showing a loss of sequence from within of UL145 up to UL148C, with concomitant insertion of E. coli DNA; (ii) a minor population showing a loss of sequence from within UL142 up to and including part of UL139. (E) In Merlin-UL128^G>T^, mutant genomes encoded a nonsynonymous single nucleotide polymorphism (purple) in UL141. (F) In Merlin-UL128L^FIX^, mutant genomes underwent an in-frame deletion (green) in UL141.

**FIG 3:**
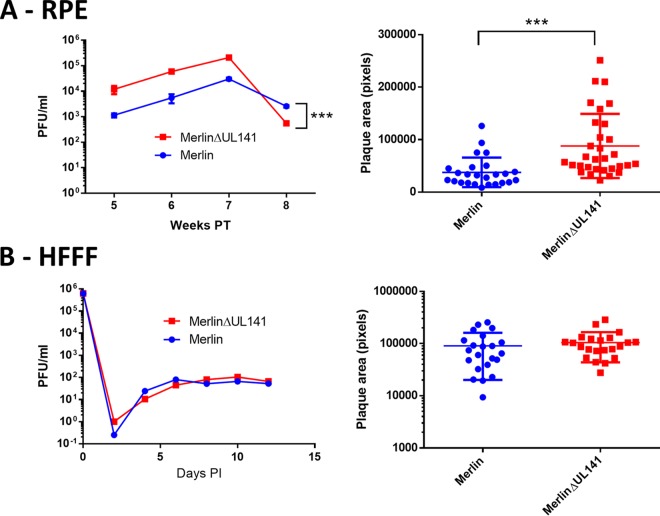
Comparison of growth of Merlin-tetUL128^wt^ (Merlin) with that of the same virus, but with UL141 deleted (Merlin ΔUL141). (A) RPE-1 cells were transfected with Merlin or with Merlin ΔUL141. At the indicated times, supernatant was titrated on HFFF cells. Alternatively, at 3 weeks posttransfection (PT), plaque sizes were measured. (B) HFFF cells were infected with Merlin or Merlin ΔUL141. At the indicated times, supernatant was titrated on HFFF cells. Alternatively, at 2 weeks posttransfection, plaque sizes were measured. Error bars show standard errors of the means; data were analyzed by two-way analysis of variance (cell-free titers) or *t* test (plaques sizes). PI, postinfection.

All Merlin variants were produced by using a self-excising vector system (15), and mutations were not associated with the region from which the vector had been excised. However, other mutations occurred. UL128L was stable within the Merlin viruses, except for Merlin-UL128L^wt^, in which a small minority (<3%) of the population contained a deletion of UL128 to UL136, replaced by a sequence derived from US20 flanked by E. coli sequences (Fig. 2). Indeed, a range of deletions and mutations in the U_L_/*b*′ region (UL148 to UL150) affected nearly all Merlin-based viruses (Table 2; Fig. 2). Thus, Merlin-UL128L^3301^ suffered a deletion of UL147 to UL150 that was compensated by duplication of RL1 to RL12 (Fig. 2C), Merlin-UL128L^TB40^ consisted primarily of a deletion of UL145 to UL148C (replaced by E. coli DNA) and a low-abundance deletion of UL142 to UL139 (Fig. 2D), Merlin-UL128L^FIX^ exhibited a mutation in UL141 (Fig. 2E) in the majority of genomes, and Merlin-UL128^G>T^ had a UL141 deletion (Fig. 2F). Thus, mutations affecting UL141 were repeatedly identified in Merlin-based viruses during passage in RPE-1 cells. To determine whether this was directly due to UL141, UL141 was deleted from Merlin-tetUL128^wt^ (Merlin ΔUL141), and both cell-free titers and plaque sizes were measured following infection of epithelial cells (Fig. 3A) or HFFF (Fig. 3B). Both titers and plaque sizes were increased when virus lacking UL141 was grown in RPE-1 cells but not in HFFFs. Thus, loss of UL141 provides a selective advantage in RPE-1 cells.

Mutations outside the U_L_/*b*′ region were identified in some instances and included amino acid substitutions affecting UL26, UL75, and US8 (Table 2). Merlin-UL130^A>G^ was unusual in maintaining its genomic integrity in the short-term culture involved in this experiment. However, this virus was recovered only once in RPE-1 cells. Given that UL141 restricts virus growth in RPE-1 cells (Fig. 3) and that all other Merlin viruses experienced mutations in U_L_/*b*′, it seems likely that mutations in the U_L_/*b*′ genome region of Merlin-UL130^A>G^ would also be selected upon further culture.

In summary, although it is possible to conserve the sequence and function of UL128L by culturing HCMV in epithelial cells, almost all viruses assessed (7/8) acquired mutations elsewhere in the genome, in particular, around the site of the vector (if it was retained following generation of virus) or in U_L_/*b*′, centered on UL141. This general instability undermines confidence in the use of this cell type for extended HCMV propagation.

### Stability of BAC-derived HCMV strains in fibroblasts.

Since passage of HCMV in epithelial cells was consistently associated with mutation, all BACs (Table 1) were transfected into fibroblasts, and virus was allowed to spread through the monolayers. Cell-free virus was then collected and used to infect a fresh monolayer at a low MOI, and virus was allowed to spread. This was repeated five times before viral DNA was sequenced. In this manner, samples archived at each passage could be used to track when mutations emerged by retrospective PCR amplification and sequencing. In each case in which a mutation was observed during generation of virus, the mutation was not detected in the parental BAC.

**FIG 4:**
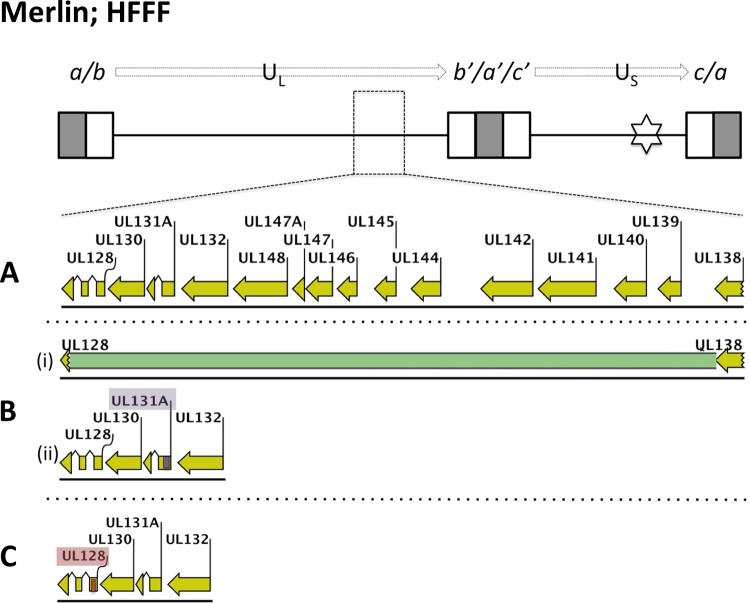
Mutant populations detected in viruses derived from Merlin-BAC variants containing wild-type UL128L ORFs from strain Merlin or from the unpassaged strain 3301 following five sequential passages in HFFFs. A schematic of the BAC-cloned Merlin genome, as previously described (Fig. 2), is shown at the top of the figure. (A) Layout of ORFs UL128 to UL138 in parental Merlin-BAC constructs. (B) Mutant populations detected in Me-UL128L^wt^: (i) a major population underwent deletion (green) of sequence from within UL128 and ending in the UL138/UL139 intergenic region; (ii) a minor mutant population underwent a frame-shifting 4-bp deletion (purple) in UL131A. (C) In Merlin-UL128L^3301^, mutant genomes acquired a frame-shifting insertion of a G residue, as well as a single nucleotide substitution (C>T) (red) at a neighboring residue position, in UL128 exon 1.

The mutation in the BAC used to generate Merlin-UL128L^mut^ was selected naturally when Merlin was first isolated from the clinical sample. In two independent transfection experiments, the sequence of Merlin-UL128L^mut^ at passage 5 exactly matched that of the parental BAC (minus the vector). However, when strain Merlin contained a wild-type UL128L, either from Merlin itself (Merlin-UL128L^wt^) or the unpassaged strain 3301 (Merlin-UL128^3301^), the majority of genomes suffered mutations involving UL128L. The major population in Merlin-UL128^wt^ contained a deletion extending from UL128 to UL139 (Table 3 and Fig. 4B, part i), and a second population contained a frameshift in UL131A (Fig. 4B, part ii). In Merlin-UL128L^3301^, a frameshift was observed in UL128 (Fig. 4C), as well as a substitution in the region between UL54 and UL55. Retrospective investigation detected each of these genetic changes at passage 1.

**TABLE 3:** Mutant populations viruses generated by 5 passages in fibroblast (HFFF) cells

Virus	Mutation location (nt)^*a*^	ORF(s) affected	Mutation and/or coding effect	Mutant population (% of sequenced population)
Merlin-UL128L^mut^			No *de novo* mutations detected	
Merlin-UL128L^wt^	176320–187358	UL128–UL139	11,039-bp deletion	97
	178063–178066	UL131A	4-bp deletion; frameshift	3
Merlin-UL128L^3301^	228878–446	UL128 exon 1	G insertion; frameshift	64
	81884	UL54/UL55 IG	G>A substitution	
TR	194202-BAC	*a*′-US3	6,087-bp deletion: 3,899 bp from *a*′ to the BAC vector, plus 2,188 bp of the BAC	92
			4,306-bp deletion: 3,899 bp from *a*′ to the BAC vector, plus 407 bp of the BAC	92
TB40-BAC4	228878–446	US7	Deletion of 173 bp from *a*′ to the BAC vector, entire BAC vector, and 446 bp into US7	6
FIX	228002–60		7,650-bp deletion; sequence from *a*′ to BAC vector plus 7 bp	100
Merlin-UL128L^TB40^	177130–179950	UL130–UL148	2,807-bp deletion	54
	NA	eGFP	T>A substitution	61
Merlin-UL128^G>T^	61647	UL47	G>C (D>H) substitution	29
	83915	UL55	T>A (D>V) substitution	11
Merlin-UL128L^FIX^			No *de novo* mutations detected	
Merlin-UL130^A>G^	NA	eGFP	720-bp deletion	99

a Locations are based on the sequences of TR-BAC (GenBank accession number AC146906.1), TB40-BAC4 (GenBank EF999921.1), and Merlin-BAC (GenBank GU179001.1). The TR-BAC, TB40-BAC4, and FIX-BAC sequences deposited in GenBank commence immediately following the site of vector insertion, and coordinates of mutations in viruses derived from these clones describe sequences either side of the BAC vector. NA, not available; nt, nucleotide.

**FIG 5:**
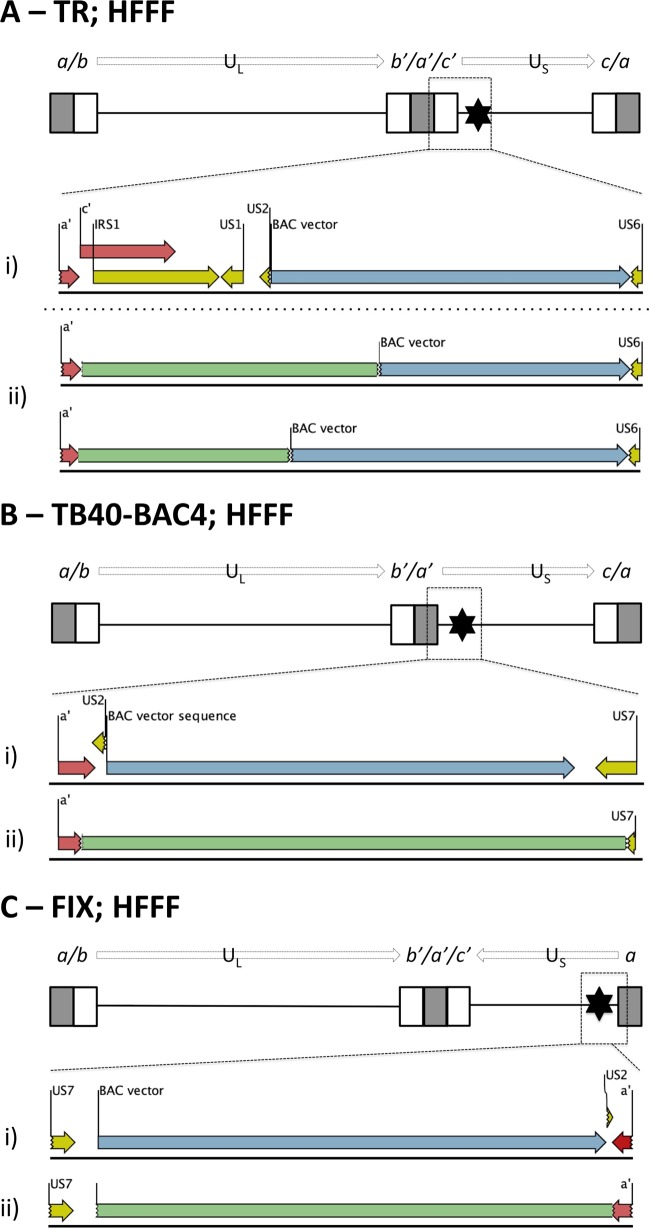
Mutant populations detected in viruses derived from TR-BAC, TB40-BAC4, and FIX-BAC following five sequential passages in HFFFs. (A) Mutant populations detected in TR virus. A schematic of TR-BAC, as previously described (Fig. 1), is shown at the top of the panel. The junction of the short internal repeat (IR_S_) and U_S_ regions in the parental TR-BAC is shown, including part of *a*′ and *c*′ sequence repeats (red), ORFs IRS1 and US1, and remaining fragments of US2 and US6, disrupted by insertion of the BAC vector sequence (blue). Sequences deleted (green) in mutated TR genomes, originating from adjacent to the *a*′ sequence and up to different positions in the BAC vector, are indicated (ii). (B) Mutant populations detected in TB40-BAC4 virus. A schematic of TB40-BAC4 genome, as previously described (Fig. 1), is shown at the top of the panel. The junction of the short internal repeat and U_S_ regions in the parental TB40-BAC, including the *a* sequence (red) fused to part of US2, the BAC vector sequence (blue), and US7, is shown (i). Sequence deleted (green) in mutated TB40-BAC4 genomes, originating from within the *a*′ sequence, encompassing the BAC vector, and disrupting US7, is indicated (ii). (C) Mutant populations detected in virus from FIX-BAC. A schematic of the parental FIX-BAC is shown at the top of the panel. ORFs in the U_S_ genome region are inverted compared to those in the U_L_ genome region, and the genome lacks a terminal *c* sequence in the short terminal repeat genome region. The junction of the short terminal repeat and U_S_ regions in the parental FIX-BAC genome, including US7, the BAC vector sequence (gray), the remaining part of US2 that is fused to the terminal *a* sequence (red) in the short terminal repeat region, is shown (i). Sequence deleted (green) in mutated FIX genomes, encompassing the BAC vector sequence and up to within the *a* sequence, is indicated (ii).

Following serial passage in fibroblasts, UL128L sequences in strains TR, TB40, and FIX all matched exactly the corresponding sequences in the parental BACs. However, all three strains exhibited substantial deletions elsewhere in the genome, which were reminiscent of the rearrangements observed during propagation of viruses derived from the same BACs in epithelial cells (Table 3). In TR, two distinct mutations were detected, both comprising the deletion of a sequence extending from the *a*′ region to a site within the vector (Fig. 5A). In TB40-BAC4, virus lacked sequence extending from within *a*′, across the entire vector, into US7 (Fig. 5B). In FIX, a deletion was observed extending from immediately outside the terminal *a*′ sequence, across the entire vector, into US2 (Fig. 5C). Thus, in all three strains, mutations were linked to the vector, and these were selected rapidly, being detected at passage 1.

**FIG 6:**
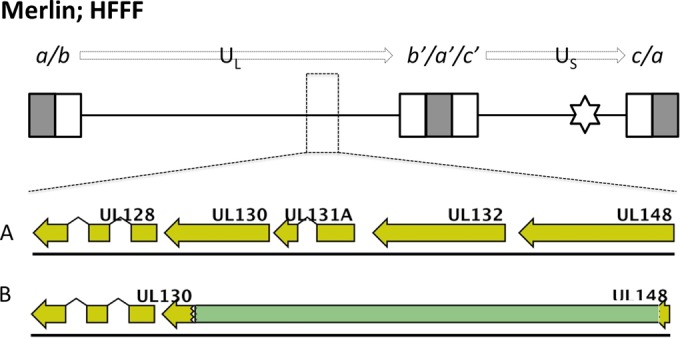
Mutant populations in stocks of Merlin variants containing UL128L ORFs from TB40-BAC4 following five passages in HFFFs. Schematic of the BAC-cloned Merlin genome as described previously (Fig. 2). (A) ORFs UL128 to UL148 in parental Merlin-UL128L^TB40^. (B) In Merlin-UL128L^TB40^ at passage 5 in HFFFs, sequence was deleted from within UL130 and ending in UL148.

Wild-type UL128L represses the growth of Merlin in fibroblasts, and mutations are selected rapidly (12, 15, 23, 24) (see above). When the entire UL128L region or the previously identified single nucleotide substitutions in UL128L of TB40-BAC4 or FIX-BAC were inserted into the Merlin genome, repression of virus spread and release in fibroblasts were less pronounced (23). In accord with this finding, only one of the four Merlin-based viruses containing UL128L sequences from these BACs (Merlin-UL128L^TB40^, Merlin-UL128L^FIX^, Merlin-UL128^G>T^, and Merlin-UL130^A>G^) acquired mutations in UL128L, with Merlin-UL128L^TB40^ experiencing the loss of UL130 to UL148 by passage 1 (Fig. 6). Mutations were observed elsewhere in the genomes of three of these viruses, but in two of these they were associated with the eGFP reporter gene: a T>A substitution in Merlin-UL128L^TB40^ and a deletion near the 3′ end of the gene in Merlin-UL130^A>G^ at passage 2. Only one of these viruses contained mutations in genes outside UL128L and the reporter gene, with Merlin-UL128^G>T^ acquiring amino acid substitutions in UL47 and UL55 by passage 4.

Thus, viruses derived from TR-BAC, TB40-BAC4, and FIX-BAC lack a specific mechanism to excise the vector in eukaryotic cells and consistently developed mutations within or adjacent to the vector in fibroblast cells. Merlin-BAC was designed to self-excise the vector in mammalian cells (57, 58), and this eliminated the problem. As a result, viruses derived from Merlin-BAC were stable during multiple passages as long as expression of UL128L was impaired by suitable mutations introduced into the BAC. Variants that contained reduced levels of the pentameric complex in the virion (due to the presence of nucleotides derived from TB40-BAC4 or FIX-BAC) acquired mutations in UL128L mutations only rarely. However, mutations were occasionally detected elsewhere in the genome.

### Repression of RL13 and UL128L enables stable passage of viruses containing the complete, wild-type HCMV gene complement in fibroblasts.

In situations in which expression of the pentameric complex was ablated or substantially reduced by mutation of UL128L, viruses derived from Merlin-BAC only rarely acquired *de novo* mutations during passage in fibroblasts (above). The Merlin-UL128L^mut^ BAC (lacking RL13 and UL128) has thus far been used to generate 44 different HCMV recombinants or mutants, and their genomes have been sequenced (30, 59). A total of 40 viruses had genome sequences that were identical to the sequence of the parental BAC (minus the vector), and only four were mutated (Table 4). These mutations include deletion of viral genes, incorrect excision of the vector, and incorporation of an E. coli insertion element. Thus, although these viruses were generally stable, apparently random mutations occurred in about 10% of viruses following passage *in vitro*.

**TABLE 4:** Mutations in 4/44 Merlin-UL128L^mut^-derived viruses following passage in fibroblasts

Location of mutation (nt)^*a*^	ORF(s) affected	Mutation and/or coding effect	Mutant population (% of sequenced population)
29048	UL23–UL24, intergenic	C>T	55
29531–31054	UL23–UL24	1,504-bp deletion	64
BAC-240244	US29–US34A	2,215 nt of the left-hand end of the BAC vector remains; there is a 72-nt duplication of a BAC sequence, then a deletion encompassing the remainder of the BAC cassette, and a deletion of 5,076 nt of the HCMV coding sequence to the right of the BAC	100
18049–18050	UL10	1,338-bp insertion (1,329 bp of *E*. coli sequence plus 9 bp of duplicated viral sequence)	76
206155–206156	US11	1,190-bp insertion of E. coli IS*150*, plus a 3-nt duplication of viral sequence	100
206155–206156	US11	1,190-bp insertion of E. coli IS*150* (opposite orientation to above)	100

a Relative to Merlin-BAC (GenBank accession number GU179001.1). nt, nucleotide.

Although viruses lacking RL13 and UL128L proved to be generally stable and although they have been used in many studies because of this property, the inability of these viruses to infect cell types relevant to intra- and interhost dissemination (e.g., epithelial cell, endothelial cell, myeloid cell types), as well as the altered growth phenotypes they display compared to the growth phenotype of clinical virus (i.e., a predisposition for cell-free virus spread, as opposed to cell-to-cell spread), means that it is desirable to be able to generate and passage viruses retaining wild-type versions of RL13 and UL128L (44, 60–72). Previously, we exploited a Tet-regulated system to repress expression of RL13 and UL128L during generation of virus from a BAC (15). In the present study, we hypothesized that Tet repression of RL13 and UL128L would enable virus encoding the complete, wild-type gene complement to be propagated stably and efficiently *in vitro*. To test this, binding sequences for the Tet operator were inserted upstream of UL131A in the Merlin-UL128L^wt^ (Merlin-tetUL128L^wt^), Merlin-UL128^G>T^ (Merlin-tetUL128^G>T^), and Merlin-UL130^A>G^ (Merlin-tetUL130^A>G^) BACs. Tet operators were also inserted upstream of a repaired RL13 gene in a virus either lacking UL128 (Merlin-tetRL13^wt^) or containing UL128 (Merlin-tetRL13^wt^/tetUL128L^wt^). Viral stocks were generated from all BACs in HFFF-Tet cells. The genome sequences of single viruses (Merlin-tetUL128^G>T^ and Merlin-tetUL130^A>G^) or duplicate viruses (Merlin-tetUL128^wt^, Merlin-tetRL13^wt^, and Merlin-tetUL128L^wt^/tetRL13^wt^) independently derived from each BAC were identical to those of the parental BACs (minus the vector). Thus, repression of RL13 and UL128L enabled the passage of virus containing the complete gene complement of HCMV, with minimal risk of mutations being acquired or selected *de novo*.

## DISCUSSION

The capacity of clinical HCMV strains to accumulate mutations rapidly and progressively during propagation *in vitro* has resulted in research being conducted on viruses that differ from strains circulating *in vivo* in terms of their genetic content and in terms of important phenotypes such as tropism, immunomodulation, cell-associated dissemination, and virulence (12, 14, 41, 44, 73). The advent of BAC technology has provided a potential way around this problem by facilitating access to a stable, characterized, clonal source of the viral genome. However, there are problems with this approach that need to be addressed. In order to generate a BAC, a clinical HCMV strain is normally isolated and expanded *in vitro* as a prelude to cloning the genome into a BAC vector, and the virus is likely to mutate during this process. This risk can be monitored by comparing the sequence of the BAC-cloned genome with that of the original clinical sample, carrying out repairs as required. Without this comparison, there is no guarantee that mutations will be identified since these may be subtle in their effects (e.g., they may cause amino acid replacements or affect noncoding functions) (12, 23, 44, 49, 74). Furthermore, even if the sequences of the clinical virus and the BAC-cloned genome match exactly, there is a possibility that mutations will be acquired when virus is generated from the BAC, as we have demonstrated previously (15) and in the present study. This risk can be assessed by sequencing the genome of the virus generated. As a result of exploring this double jeopardy and identifying potential control points, we have identified a method for propagating HCMV through multiple serial passages while retaining an intact complement of genes.

TR-BAC, TB40-BAC4, and FIX-BAC are used widely and contain intact protein-coding regions in UL128L. We have demonstrated that, during passage of the viruses derived from these BACs in fibroblasts, mutations are less likely to be selected in UL128L than when virus derived from Merlin-BAC is passaged. In the cases of TB40-BAC4 and FIX-BAC, this property likely results from subtle mutations in UL128 and UL130, respectively, which we have shown previously reduce the level of UL128L proteins in the virion, thereby reducing the inhibitory impact of UL128L on viral growth in fibroblasts (23). Genetic differences outside UL128L may also contribute to the stability of UL128L in these strains since no mutations were selected in UL128L of TR, FIX, or TB40 following generation in fibroblasts, and yet when UL128L sequences from TB40-BAC4 were placed into Merlin-BAC (Merlin-UL128^TB40^), mutations in UL128L were selected in one of the two viruses analyzed. Similarly, we previously observed the selection of mutations in TR UL128L when it was placed into the Merlin genome (23).

Although mutations in UL128L were not selected when virus was derived from TR-BAC, TB40-BAC4, or FIX-BAC, *de novo* mutations were repeatedly selected in the unique short (U_S_) region (in association with the vector) whether viruses were propagated in epithelial or fibroblast cells. Mutations affecting the genes in this region were not observed during a previous study when viruses derived from clinical material (rather than BACs) were passaged in cell culture (12). Thus, it is possible that the selection pressure results from an increase in genome size caused by the presence of the vector, coupled with genome size limitations operating during virion morphogenesis (57, 75). Indeed, the length of TR-BAC is appreciably larger (241,327 bp) than that of the original TR virus (235,029 bp when circularized) although FIX-BAC (235,597 bp) is only marginally larger than the parental strain VR1814 (234,614 bp when circularized). Alternatively, the selection pressure may be due to an incompatibility between part of the U_S_ region and the vector that impacts negatively on viral replication *in vitro*. Irrespective of the reasons, reengineering these BACs to contain a self-excising BAC cassette, as has been done for TR (76), is likely to offer a solution to these problems. In fact, based on the general scarcity of mutations observed elsewhere in the genomes of TR-BAC, FIX-BAC, and TB40-BAC4 in this study, such a construct may be quite stable in culture. However, it should be appreciated that without the sequence of the original clinical material, it is impossible to know how much this stability derives from mutations that have already occurred (prior to BAC cloning) (15, 23, 44, 49, 50).

Interestingly, mutations affecting UL84 were seen in viruses derived from both TR-BAC and TB40-BAC4 in RPE cells. This open reading frame (ORF) encodes a multifunctional protein with activities to both promote and impede virus replication (77). In both TR and TB40-BAC4, UL84 is nonessential for growth (78, 79). The N-terminal residues of UL84 interact with UL44 and are required for genome replication (80), while both the N- and C-terminal regions are involved in transcriptional repression via interactions with ppUL122 (77). The mutations acquired by TR and TB40-BAC4-derived viruses each resulted in the loss of C-terminal residues of pUL84. Since the mutants made up ∼80% of the sequenced population, loss of domains involved in the IE2-mediated repressive activity of UL84 may result in a growth advantage *in vitro*.

No mutations were observed in the region of the Merlin genome that contains the residual *loxP* site following vector self-excision. However, mutations in the U_L_/*b*′ region were frequently selected when viruses were generated in RPE-1 cells. Comparable genetic changes were observed occasionally in our previous study when clinical HCMV strains were passaged *in vitro* without the involvement of BACs (12). However, this was after at least 32 weeks of propagation, whereas, in the current study, mutations appear to have been selected much more rapidly. This may reflect the different ways in which viruses were grown in the two studies. In the previous study, cultures were small and were split weekly so that the number of infected cells remained low. In the present study, cultures were grown until complete infection was observed and then expanded to include a much greater number of infected cells before cell-free virus was harvested. It is notable that all of the mutations in the U_L_/*b*′ region detected in viruses generated from Merlin BACs in epithelial cells involved the loss of UL141, with some viruses showing mutation of this gene alone. This confirms and extends the observations from our previous study, in which we reported that the region of UL145 to UL140 was prone to mutation during passage of clinical HCMV strains *in vitro*, albeit that previously loss of this gene region occurred in both fibroblast and epithelial cells (12). Thus, in addition to being an immune evasion gene (28, 29, 31), UL141 is inhibitory to the growth of HCMV in epithelial cell culture, with selection against UL141 presumably resulting in loss of adjacent genes in U_L_/*b*′ in this cell type. Since UL141 downregulates levels of the proteins CD155, CD112, and TrailR2 from the infected cell surface (28, 29, 31), fluorescence-activated cell sorting (FACS) analysis could be used to determine whether mutations involving UL141 have occurred in any given virus stock. UL141 is also mutated in TB40-BAC4 (47), whereas neither TR-BAC- nor FIX-BAC-derived viruses suffered deletions in the U_L_/*b*′ region during passage in epithelial cells. This difference could be due to the acquisition of subtle, unrecognized mutations in this region, similar to those previously identified in RL13 and UL128L of TB40-BAC4 and FIX (23), or it may indicate genuine strain-specific differences. Identifying strain-specific phenotypes, without the confounding effects of *in vitro* mutations, will require the construction of validated BAC-cloned HCMV genomes from independent HCMV clinical isolates (44).

Another interesting observation was that in one virus (Merlin-UL128^wt^), a minority population of mutants in UL128L was selected despite this genome region being required for efficient infection in RPE-1 cells. Presumably, this could only happen if the genome lacking UL128L was complemented by coinfection with a wild-type genome. All viruses that suffered large deletions during passage in RPE-1 cells contained additional sequence in place of the U_L_/*b*′ region, either viral or E. coli DNA. Deep sequencing detected E. coli DNA in DNA preparations for transfection; presumably this was the source of DNA that subsequently recombined with the virus genome during passage. Nevertheless, although growing viruses in RPE-1 cells generally prevents selection of mutations in UL128L, the frequent selection of mutations in the U_L_/*b*′ region necessitates the exercise of caution in using these cells for extended passage of HCMV.

Whereas the mutations that occurred in viruses derived from Merlin BACs in epithelial cells tended to be localized to the U_L_/*b*′ region, those in fibroblasts tended to occur in UL128L, with the likelihood of mutation correlating with the parental UL128L sequence. Thus, whereas viruses containing wild-type UL128L suffered from mutations involving UL128L, viruses expressing reduced levels of the pentameric complex due to the insertion of nucleotides from TB40-BAC4 or FIX-BAC UL128L (23) exhibited such mutations less commonly (1/4 viruses). Moreover, viruses in which UL128L expression was completely ablated by mutation, or reduced by Tet repression, did not mutate in UL128L, and additional mutations elsewhere in the genome were rare (4/54 viruses). The effects of the rare mutations outside UL128L ranged from amino acid sequence changes in important immunological targets (e.g., UL55, encoding glycoprotein B [gB]) to loss of multiple genes (e.g., US29 to US34A) (Table 4). In some cases, these mutations occurred during passage of BAC-derived virus, although in two cases the acquisition of a transposon apparently occurred in E. coli. The use of bacterial strains that have been engineered to remove mobile DNA may help solve the problem of transposon insertion (81), while alternative methods of introducing BAC DNA into cells, such as invasive bacteria (82) or adenofection (83), may reduce the chances of mutations being generated during transfection.

Nevertheless, these apparently random mutations, and the mutations seen around the vector in viruses generated from TR-BAC, TB40-BAC4, and FIX-BAC, raise an important issue. Viruses derived from HCMV BACs are used widely, often to study the effects of specific genetic changes (e.g., deletion of a gene of interest). It is generally assumed that the only substantive difference between the parental BAC and the generated virus will be the intended modification. Revertants are often produced to control off-target mutations, involving repair of the modified sequence within the BAC. This type of control may suffice for identifying alterations introduced unintentionally during the process of BAC modification, but it is inadequate for monitoring unanticipated changes arising after the BAC is transfected into eukaryotic cells. It is possible to safeguard against this risk by deriving and analyzing multiple viruses from the same BAC. However, the sequencing of the viral stocks to be used in experiments offers a more robust, informative, and economical method for validating genomic integrity.

In this study, whole-genome sequencing of BAC-derived viral genomes enabled us to identify methods to passage HCMV genomes stably *in vitro*. Infectious HCMV BACs provide an excellent source of fully characterized genomic DNA and clonal virus. We have learned that propagation of BAC-derived virus in epithelial cells should be performed with caution since it can lead to the rapid selection of mutations involving UL141. Where there is a need to propagate a virus containing the complete, wild-type genome of an HCMV strain, the strategy that results in the least chance of unwanted mutations being selected is to derive virus from a BAC containing a self-excising vector, to passage the virus in fibroblasts, and to repress the UL128L and RL13 functions. Repression can be relieved subsequently by infecting HFFF cells that do not express the Tet repressor or by adding doxycycline, thus enabling experiments to be performed using virus expressing the complete gene complement of wild-type HCMV. Clinical strains consistently acquire mutations in UL128L during passage in fibroblasts (12), and BAC-cloned strains (e.g., TB40-BAC4 and FIX) that do not acquire ablative mutations in UL128L during culture tend to have preexisting mutations that result in smaller amounts of pUL128L being incorporated into the virion (23). Thus, it seems likely that repression of UL128L (and RL13) will be required for the stable passage of any virus where the genome sequence matches that of a clinical strain.

## Supplementary Material

Supplemental material
