# Supplementary material for: Genetic Stability of Bacterial Artificial Chromosome-Derived Human Cytomegalovirus during Culture In Vitro
Source: J Virol. 2016 Mar 28;90(8):3929–43. doi: 10.1128/JVI.02858-15 (PMC4810542; doi:10.1128/JVI.02858-15)
Supplement: Supplemental material [file supp_90_8_3929__index.html]

Genetic Stability of Bacterial Artificial Chromosome-Derived Human Cytomegalovirus during Culture In Vitro — Supplemental material 

# Genetic Stability of Bacterial Artificial Chromosome-Derived Human Cytomegalovirus during Culture *In Vitro*

## Supplemental material

- Supplemental file 1 -

  Table S1 (Primer pairs used for PCR-based sequencing analysis of mutations acquired by viruses following 5 sequential passages in fibroblasts.)

  PDF, 182K
