# Supplementary material for: Genetic Stability of Bacterial Artificial Chromosome-Derived Human Cytomegalovirus during Culture In Vitro
Source: J Virol. 2016 Mar 28;90(8):3929–43. doi: 10.1128/JVI.02858-15 (PMC4810542; doi:10.1128/JVI.02858-15)
Supplement: Supplemental material [file JVI.02858-15_zjv999091533so1.pdf]

Supplementary Table 1. Primer pairs used for PCR based sequencing analysis of mutations acquired by viruses following 5 sequential passages in fibroblasts.

Virus	Mutation	Primer Forward	Co-ordinates[a]	Primer Reverse	Co-ordinates[b]
TR	Δ 6,087 bp from *a′* into BAC vector	ACC GCT TCC TCT TTT CCC	230,704-230,721	ACT CTC CTG CCT TTC TAT CTT	n/a
	Δ 4,306 bp from *a′* into BAC vector			ACA GGC CAG AAA GAC ATA ACT	n/a
TB40-BAC4	Δ: 173 bp from *a′*- BAC vector, entire BAC vector, 446 bp into US7	CGG GGA ATC AAA CAG ACA CA	228,655-228,674	CCC CCA AAC CTA AGT CCA ACA	846-866
FIX	Δ 7,650 bp: Sequence from *a′* to BAC vector + 7 bp	CTT CCT CTT TTC CGC GTC TT	33,004-33,023	CCT CCT CCG TTC CGT ATT CT	184-203
Me-UL128L^{wt}	Δ 11,039 bp from UL128 – UL139	CCA GCC GTT TGT GTT TCT T	176,173-176,191	GGT TCA TCG TCT TCG TCG T	187,545-187,563
	Δ 4 bp in UL131A exon 1	TTC CCG TTG TCA TGC ATC T	177,824-177,842	ACC ATC GAA TCT CAC CTC T	178,362-178,380
Me-UL128L^{3301}	Insertion G, substitution C>T in UL128 exon 1	CAC CAC AGA ACG CAG ACA A	176,695-176,713	AAC CAA ACC ATC CTC CAA C	177,208-177,226
	Substitution G>A in UL54-UL55 Intergenic region	CAA GGA CAC GGC CAA ACC A	81,612-81,630	ACG CAG GAC AAG GGA CAG A	82,098-82,116
Me-UL128L^{TB40}	Δ 2,807 bp in UL130-UL148	TCT GGT TAT TGG CCT CGG T	176,971-176,989	ATT CCT GCT CCT CAT CTT CT	180,229-180,248
	Substitution T>A in eGFP	GCT TCT CGT TGG GGT CTT T	n/a	CCG GTG TGC GTT TGT CTA T	n/a
Me-UL128$^{G>T}$	Substitution: G>C in UL47	TAA TGT AGA GGT GTC GCG G	61,381-61.399	TTC GGC TAT TAC GGT CTG G	62,030-62,048
	Substitution: T>A in UL55	TCA AGA AAA GCC ACC AAC C	83,762-83,780	TTC CAA CAC CCA CAG TAC C	84,067-84,085
Me-UL130$^{A>G}$	Δ 720 bp in eGFP	GCT TCT CGT TGG GGT CTT T	n/a	CCG GTG TGC GTT TGT CTA T	n/a

[a] + [b] – Primer binding region co-ordinates are given relative to sequences of BAC-cloned strains deposited in GenBank: Merlin (GU179001.1) for all Merlin variants; TR (AC146906.1); TB40-BAC4 (EF2999921.1); FIX (AC146907.1). Where n/a, primers bound regions within the BAC-vector or eGFP cassette not included in the sequences deposited in GenBank. The sequences of strains TR-BAC, TB40-BAC4, and FIX-BAC deposited in GenBank commence immediately following the site of BAC vector insertion. The positions of mutations in viruses derived from these clones include sequence at each end of the deposited sequences.
